# Liver Imaging Reporting and Data System (LI-RADS) v2018: differential diagnostic value of ADC values for benign and malignant nodules with moderate probability (LR-3)

**DOI:** 10.3389/fonc.2023.1186290

**Published:** 2023-08-22

**Authors:** Xue Chen, Quanyu Cai, Jinju Xia, Huan Huang, Zhaoxing Li, Kairong Song, Ningyang Jia, Wanmin Liu

**Affiliations:** ^1^ Department of Radiology, Third Affiliated Hospital of Naval Medical University, Shanghai, China; ^2^ Department of Radiology, Tongji Hospital, School of Medicine, Tongji University, Shanghai, China

**Keywords:** liver cancer, apparent diffusion coefficient, liver imaging reporting and data system, LR-3, MRI

## Abstract

**Objective:**

To evaluate the usefulness of the apparent diffusion coefficient (ADC) in differentiating between benign and malignant LR-3 lesions classified by Liver Imaging Reporting and Data System 2018 (LI-RADS v2018).

**Methods:**

Retrospectively analyzed 88 patients with liver nodules confirmed by pathology and classified as LR-3 by LI-RADS. All patients underwent preoperative contrast-enhanced MR examination, and the following patient-related imaging features were collected: tumor size,nonrim APHE, nonperipheral “washout”, enhancing “capsule”, mild-moderate T2 hyperintensity, fat in mass, restricted diffusion, and nodule-in-nodule architecture. We performed ROC analysis and calculated the sensitivity and specificity.

**Results:**

A total of 122 lesions were found in 88 patients, with 68 benign and 54 malignant lesions. The mean ADC value for malignant and benign lesions were 1.01 ± 0.15 × 10^3^ mm^2^/s and 1.41 ± 0.31 × 10^3^ mm^2^/s, respectively. The ADC value of malignant lesions was significantly lower than that of benign lesions, p < 0.0001. Compared with other imaging features, ADC values had the highest AUC (AUC = 0.909), with a sensitivity of 92.6% and a specificity of 74.1% for the differentiation of benign and malignant lesions.

**Conclusions:**

ADC values are useful for differentiating between benign and malignant liver nodules in LR-3 classification, it improves the sensitivity of LI-RADS in the diagnosis of HCC while maintaining high specificity, and we recommend including ADC values in the standard interpretation of LI-RADSv2018.

## 1 Introduction

Hepatocellular carcinoma (HCC) is the most common primary hepatic malignancy and second leading cause of cancer-related deaths worldwide, particularly in patients with cirrhosis (1). Early diagnosis of HCC in asymptomatic patients is critical to improving prognosis, and imaging techniques play a crucial role in monitoring and diagnosing HCC (2). Therefore, imaging diagnosis of HCC is essential to guide clinical diagnosis and treatment.

The American College of Radiology proposed the 2018 version of the Liver Imaging Reporting and Data System (LI-RADS v2018) as an interpretation and classification of liver observations in patients at high risk for HCC to reflect the risk of benign, malignant tumors, or HCC (3–6), with high sensitivity and specificity for HCC diagnosis (7–9). Recent literature reports have shown that the accuracy of HCC diagnosis can be further improved by adding auciliary features such as restricted diffusion, mild-moderate T2 hyperintensity, and hepatobiliary phase hypointensity (10–13). Among them, benign and malignant lesions in LR-3 classification are not easy to judge, and there are still great challenges whether patients can receive effective clinical treatment (14, 15).

Diffusion-weighted imaging (DWI) is a functional technique used to assess cellularity based on motion restriction of water molecules. DWI is currently the standard sequence for liver MR examination and is effective in detecting small liver lesions, including HCC. Compared with the surrounding liver parenchyma, HCC on DWI showed mild-to-moderate hyperintensities, which contributed to the diagnosis of HCC < 1 cm (2, 16). The analysis of DWI images can be qualitative and quantitative by apparent diffusion coefficient (ADC) maps, which measure to distinguish benign from malignant lesions and can improve the differentiation rate of benign and malignant lesions (17–19). It has recently come to light that the prognostic value of LR-3 and LR-4 classes has been investigated in the context of liver transplantation, revealing a relatively high prevalence of such observations (40.9%) among all significant nodules detected on pre-transplant imaging. Additionally, the number and size of HCC nodules play a pivotal role in determining post-transplant outcomes (20–22). In accordance with the current guidelines, short-term follow-up is recommended for nodules classified as LR-3 by LI-RADS. However, if we can more accurately determine the nature of these nodules and promptly initiate appropriate clinical interventions, including early resection for malignant lesions, we can significantly enhance the accuracy of prognosis for patients undergoing liver transplantation, ultimately ensuring their safety and well-being.

Therefore, this study focuses on whether ADC values can improve the malignant detection rate of LI-RADS v2018 classification as LR-3 nodules.

## 2 Materials and methods

### 2.1 Study participants

This retrospective study was approved by the institutional review committee and the requirement for written informed consent was waived.

From January 2020 to December 2021, a total of 1528 patients who underwent magnetic resonance imaging (MRI) and had high risk factors for HCC were identified, of whom 593 were confirmed by surgical pathology or biopsy. The MRI, clinical and histopathological data of the patients were reviewed.

**Figure 1:**
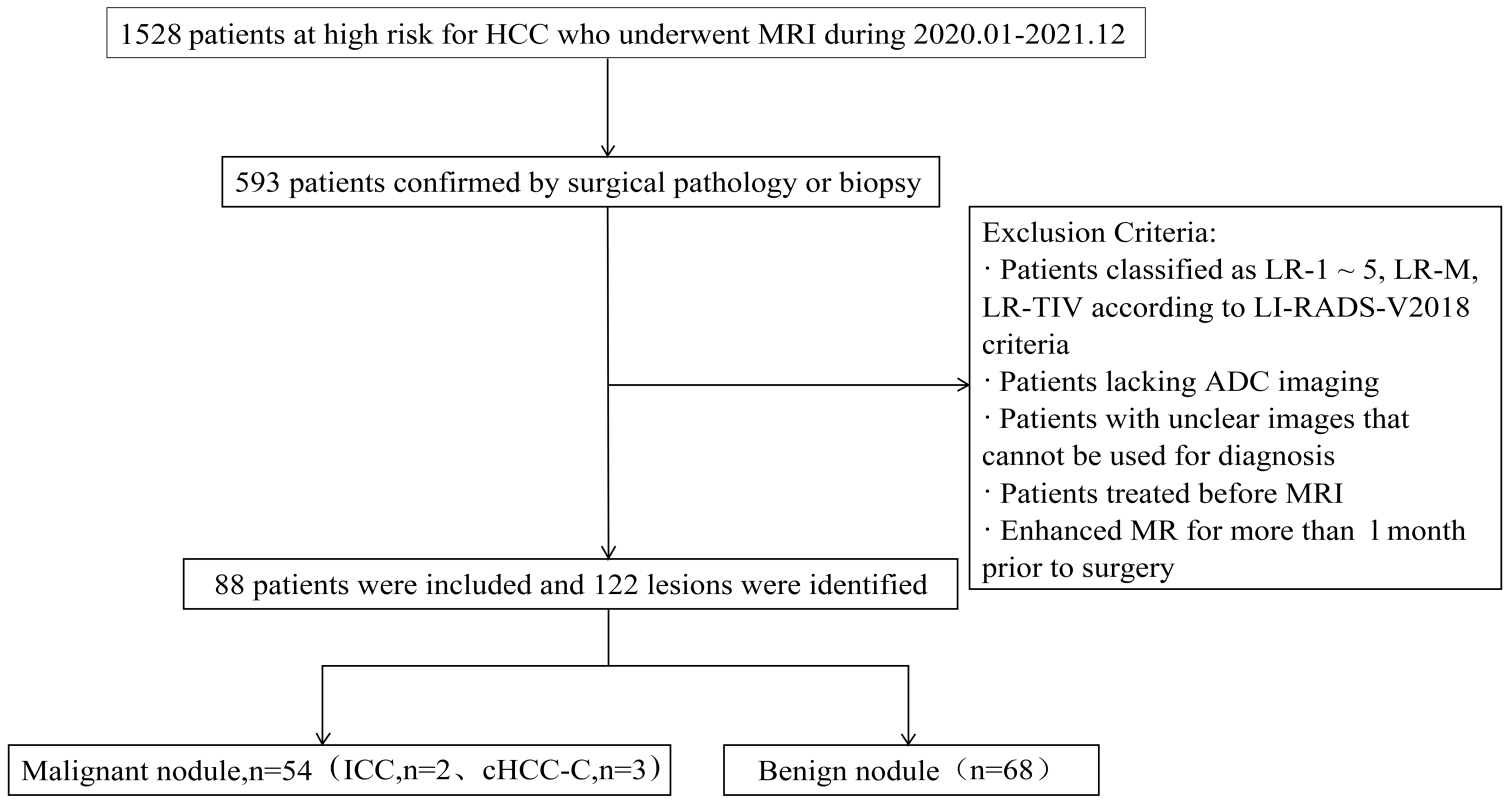
Flowchart of the study population.

The inclusion criteria were: (1) meeting the LI-RADS diagnostic criteria for the study population, including patients with cirrhosis, chronic hepatitis B infection, and previous HCC; (2) having confirmation by surgical pathology or biopsy; (3) patients underwent liver MRI enhancement and DWI scans within 1 month before surgery; and (4) having focal liver lesions classified as LR-3 lesions according to the 2018 version of the LI-RADS criteria. The exclusion criteria were: (1) previous treatment for intrahepatic lesions before undergoing MRI; (2) poor image quality precluding analysis; and (3) lack of ADC imaging (see **Figure 1** for a flow diagram).”

### 2.2 LR-3 nodule reference standards

Two experienced radiologists, with 8 and 7 years of experience respectively, independently conducted LI-RADS grading evaluations on preoperative liver nodule MRI in 593 patients. They were blinded to the patients’ pathology and clinical laboratory results. Lesions were classified according to the LI-RADSv2018 standard. Only nodules classified as LI-RADS 3 by both radiologists were included in the study.

The LI-RADSv2018 standard classifies LI-3 as follows:(1) When the nodule diameter is < 20 mm, no arterial phase enhancement (APHE), and no or only one major features (enhancing”capsule”, nonperipheral “washout”, threshold growth); (2) When the nodule diameter is ≥ 20 mm, no APHE, and no other major features; (3) When the nodule diameter is < 20 mm, nonrim APHE,and no other major features.(Threshold growth definition was simplified to: ≥ 50% size increase of a mass in ≤ 6 months) (6)

### 2.3 MRI image acquisition

Liver MRI was performed on a GE Discovery MR 750 3.0T imaging scanner. Patient fasted for 4-6 hours and were instructed to avoid deep breathing during the examination. Conventional liver MRI protocols for all patients included an axial respiratory-triggered fat-saturated T2-weighted fast spin echo sequence, respiratory-triggered diffusion-weighted imaging (b single shot echo-planar sequences with values of 0 and 800 mm^2^/s), breath-hold axial in-phase and out-of-phase T1-weighted LAVA-Flex sequences. and fat-saturated T1-weighted LAVA-Flex sequences acquired during the postcontrast dynamic (arterial, portal-venous, and delayed phases) before (noncontrast) and after contrast administration. Contrast-enhanced images were acquired following gadopentetate dimeglumine (Gd-DTPA) administration at a dose of 0.1 mmol/kg body weight. Contrast medium was injected at 2.0 mL/s using a double-headed bolus electric syringe (Spectris Solaris EP; Medrad), and arterial, portal, and delayed phase images were collected at 30 s, 60 s, and 180 s after contrast agent injection. Detailed MRI parameters are shown in **Supplementary Table 1**.

### 2.4 MR imaging analysis

Two experienced radiologists with over 8 years and 7 years, respectively, read the abdominal MRI together. They were blinded to the patients’ pathological and clinical laboratory results and only informed that the patients were at risk of HCC. The lesions were classified according to LI-RADSv2018 criteria. The interpretation results of the two physicians were initially analyzed for consistency using the Kappa value. Factors with a Kappa value greater than 0.8 were included in the final analysis. However, there were some remaining factors that showed inconsistency between the two physicians. To resolve this, a third radiologist with more than 20 years’ experience joined the review process, and ultimately, a consensus was reached among all three physicians.

Two physicians independently measured ADC values in manually defined regions of interest (ROIs) in liver nodules. Each lesion was measured three times, and the ADC values were then averaged. The measurements were taken from equal areas of the lesions while excluding adjacent liver parenchyma.

In addition, two physicians assessed the main features of imaging diagnosis and other auciliary features of HCC as defined in LI-RADS v2018, Major HCC features: (a) tumor size (diameter < 10 mm, 10 – 19 mm, ≥ 20 mm):Largest outer-edge-to-outer-edge dimension of an observation; (b) nonrim arterial phase enhancement (APHE): Nonrim-like enhancement in arterial phase unequivocally greater in whole or in part than liver. Enhancing part must be higher in attenuation or intensity than liver in arterial phase; (c) nonperipheral “washout”:Nonperipheral visually assessed temporal reduction in enhancement in whole or in part relative to composite liver tissue from earlier to later phase resulting in hypoenhancement in the extracellular phase; (d)enhancing “capsule”:Smooth, uniform, sharp border around most or all of an observation, unequivocally thicker or more conspicuous than fibrotic tissue around background nodules, and visible enhancing rim in PVP, DP, or TP. Ancillary features: (a)mild-moderate T2 hyperintensity:Intensity on T2WI mildly or moderately higher than liver and similar to or less than non-iron-overloaded spleen; (b) restricted diffusion: Intensity on DWI, not attributable solely to T2 shine-through, unequivocally higher than liver and/or ADC unequivocally lower than liver; (c)fat in mass: Excess fat within a mass, in whole or in part, relative to adjacent liver; (d) nodule-in-nodule architecture: Presence of smaller inner nodule within and having different imaging features than larger outer nodule. The definition could be found in **Supplementary Table 2** (6, 23).

### 2.5 Statistical analysis

SPSS 22.0 statistical software was utilized for data analysis. Mean and standard deviation of ADC values were computed and independent samples t-test was used to determine if ADC values could differentiate malignant from benign nodules in the LR-3 classification. ROC curve analysis was used to compare ADC values, Major HCC features, and ancillary features, to determine the area under the ROC curve (AUC), and to analyze sensitivity and specificity for HCC diagnosis in the LR-3 category. A *p*-value less than 0.05 was considered statistically significant. Delong test verified the difference between ADC values and AUC values of other auxiliary features, and P < 0.05 indicated statistical significance.

## 3 Results

### 3.1 Patient clinical data

The results showed that 88 patients were included in the study, including 72 males and 16 females, age 54.70 ± 9.8. Of these patients, 56 had cirrhosis (63.64%), 16 had chronic hepatitis B (18.18%), and 16 had HCC (18.18%). The clinical data of the patients are shown in **Table 1**.

**Table 1:** LI-RADS v2018 Clinical data of patients with liver focal lesions classified as LR-3.

Patient information	Value	
Age	54.70 ± 9.8	
Gender		
Male	81.8 (72/88)	
Female	18.2 (16/88)	
High risk factors for HCC		
Cirrhosis	63.6 (56/88)	
Chronic hepatitis B	18.2 (16/88)	
Previous HCC	18.2 (16/88)	
Tumor indicators		
AFP	+ 39.8 (35/88)	- 60.2 (53/88)
CA19-9	+ 20.5 (18/88)	- 79.5 (70/88)
PIVKA	+ 38.6 (34/88)	- 61.4 (54/88)
Malignant nodule of liver	44.3 (54/122)	
HCC	90.7 (49/54)	
ICC	3.7 (2/54)	
cHCC-CC	5.6 (3/54)	
Benign hepatic nodule	55.7 (68/122)	
DN	17.6 (12/68)	
RN	19.1 (13/68)	
FNH	4.4 (3/68)	
Hemangioma of liver	29.4 (20/68)	
Inflammatory foci in liver	20.6 (14/68)	
Hepatic adenoma	7.4 (5/68)	
Lipoma	1.5 (1/68)	

Numbers represent numbers, numbers in parenthesis represent percentages

“+” positive, “-” negative

*HCC* hepatocellular carcinoma, *ICC* intrahepatic cholangiocarcinoma, *cHC-CC* mixed liver cancer, *AFP* alpha-fetoprotein, *PIVKA* abnormal prothrombin, *DN* dysplastic nodules, *RN* regenerative nodules, *FNH* focal nodular hyperplasia of the liver.

### 3.2 Pathological classification

**Figure 2:**
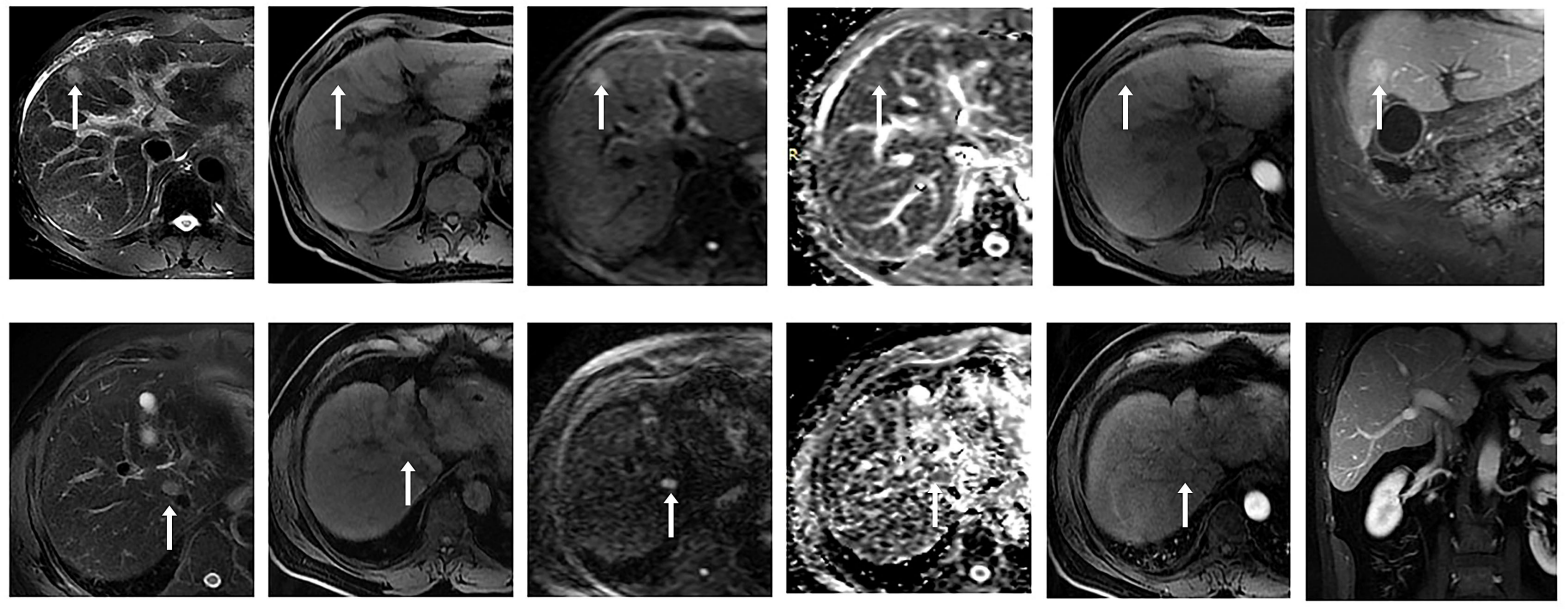
① Male, 53 years old, chronic hepatitis (B) A small nodule with a long diameter of about 1.8 cm in the left medial lobe of the liver showed high signal intensity on DWI (b value = 800 mm ²/s) and slightly high signal intensity on ADC, with a mean ADC value of about 1.409 × 10 ^-^ 3 mm ²/s. No significant enhancement was observed in the arterial phase after enhancement, and significant enhancement was observed in the portal venous and delayed phases. Postoperative pathology confirmed regenerative nodules (RN). ② A 58-year-old male patient presented with a history of chronic hepatitis B and cirrhosis. A small nodule with a long diameter of about 1.5 cm was observed at the junction of the right anterior lobe and caudate lobe of the liver, with low signal intensity on T1WI and slightly high signal intensity on T2WI. DWI (b value = 800 mm ²/s) and ADC maps showed limited diffusion, with an ADC mean value of about 0.983 × 10^-^ ³ mm ²/s. After enhancement, non-annular mild enhancement was observed in the arterial phase, isointensity was observed in the delayed phase, and no non-peripheral clearance or capsule-like enhancement was observed. Postoperative pathology confirmed hepatocellular carcinoma (HCC).

There were 135 nodules in 88 patients, according to the 2018 version of LI-RADS criteria, 13 nodules not in the LR-3 category were excluded (4 LR-1, 8 LR-2), and the remaining 122 nodules were in the LR-3 category, which were included in our study. Results analysis showed that these nodules included 54 malignant nodules (HCC, n = 49; ICC, n = 2; cHCC-CC, n = 3) and 68 benign nodules (DN, n = 12; RN, n = 13; FNH, n = 3; Hemangioma of liver, n=20; Inflammatory foci in liver, n=14; Hepatic adenoma, n=5; Lipoma, n=1). Conscientious lesions and malignant lesions assessed as LI-3 grade are shown in **Figure 2**.

Among them, malignant nodules included 49 hepatocellular carcinomas, including macrotrabecular 30, microtrabecular 11, sclerosing pattern 1, pseudoglandular pattern 1, macrotrabecular-microtrabecular 4, and macrotrabecular massive 2, intrahepatic cholangiocarcinoma 3. In addition, combined hepatocellular -cholangiocarcinoma 2, as shown in **Supplementary Table 3**.

### 3.3 Diagnostic performance analysis of imaging characteristics

**Figure 3:**
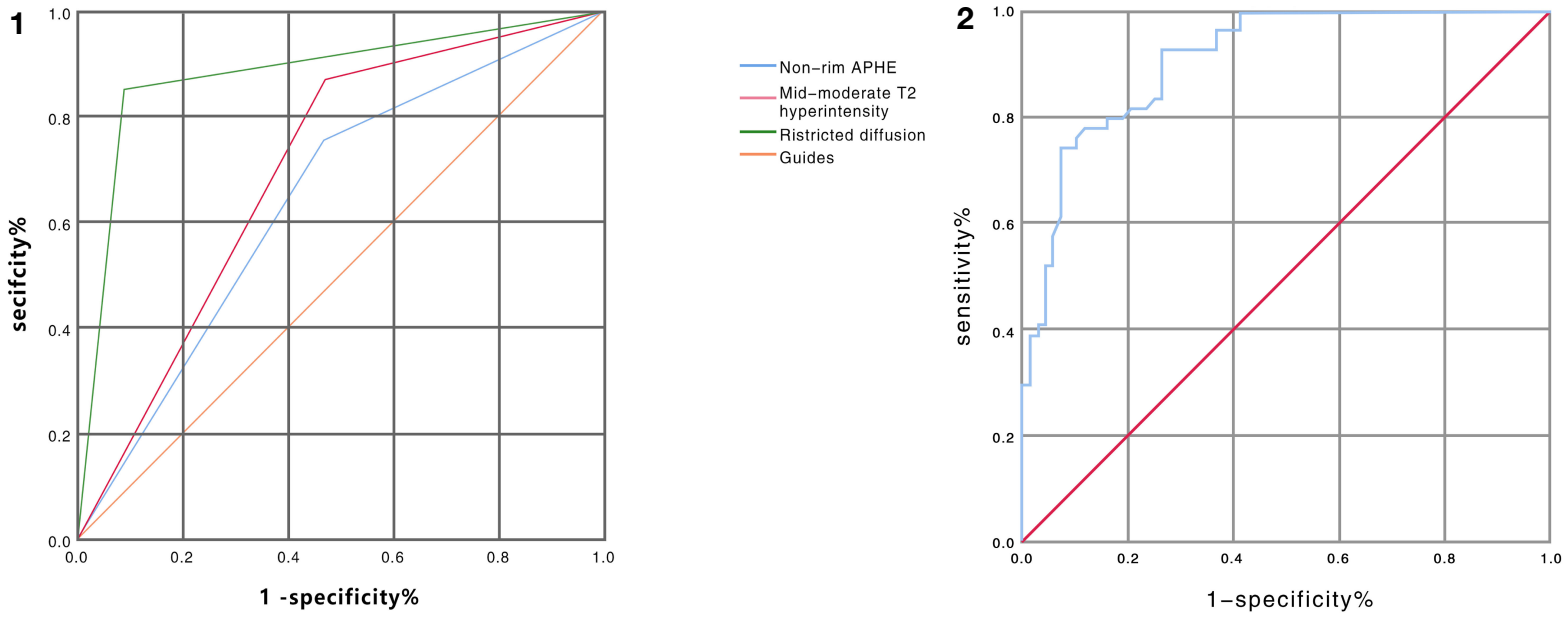
① ROC curve analysis of non-marginal APHE, mild-moderate T2 hyperintensity, and diffusion restriction in distinguishing benign from malignant nodules in LR-3 classification showed AUC values of 0.644, 0.700, and 0.882, respectively. (Blue line represents non-marginal APHE, green line represents diffusion restriction, red line represents mild-moderate T2 hyperintensity, orange represents reference line) ② ROC curve analysis of ADC values for differentiating benign from malignant nodules in LR-3 classification with AUC value of 0.909 (Note: blue line represents ADC value, red line represents reference line).

Major HCC features and ancillary features variables were selected to identify independent factors for benign and malignant nodule differentiation in LR-3 classification. ROC analysis showed that the AUC values under the curve were 0.644 [95% CI: 0.546 – 0.743], 0.700 [95% CI: 0.607 – 0.793], and 0.882 [95% CI: 0.814 – 0.949] for nonrim APHE, mild-moderate T2 hyperintensity, and restricted diffusion, respectively. P value is less than 0.05, with statistical significance, and the analysis results are shown in **Figure 3** ①. The sensitivity and specificity of differential diagnosis of non-marginal APHE were 75.9% and 52.9%. The sensitivity and specificity of differential diagnosis of mild-moderate T2 hyperintensity were 87.0% and 52.9%. The sensitivity and specificity of differential diagnosis of restricted diffusion sensitivity were 85.2% and 91.2%. Tumor size, nonperipheral “washout”, enhancing “capsule”, fat in mass, and nodule-in-nodule architecture were not significantly different in differentiating benign from malignant nodules (**Table 2**).

**Table 2:** Major HCC features and ancillary features.

Imaging features	Total (n=122)	Malignant nodule (n=54)	Benign nodule (n=68)	AUC value	P value
Major HCC features					
Tumor size					
<10mm	41.0 (50/122)	37.0 (20/54)	44.1 (30/68)		
10-19mm	57.4 (70/122)	61.1 (33/54)	54.4 (37/68)	0.536	0.496
≥20mm	1.6 (2/122)	1.9 (1/54)	1.5 (1/68)		
non-rim APHE	59.8 (73/122)	75.9 (41/54)	47.1 (32/68)	0.644	0.006^*^
non-peripheral washout	4.9 (6/122)	11.1 (6/54)	0 (0/68)	0.414	0.293
enhancing capsule	1.6 (2/122)	3.7 (2/54)	0 (0/68)	0.519	0.726
Ancillary features					
mild-moderate T2 hypersignal	70.5 (86/122)	94.4 (51/54)	51.5 (35/68)	0.700	<0.0001^*^
restricted diffusion	41.8 (51/122)	85.2 (46/54)	7.4 (5/68)	0.882	<0.0001^*^
Fat in mass	1.6 (2/122)	1.9 (1/54)	1.5 (1/68)	0.502	0.971
nodule-in-nodule appearance	0.8 (1/122)	1.9 (1/54)	0 (0/68)	0.509	0.861

Data in tables are percentages and numbers in parenthesis are numerator and denominator.

* represents a p-value less than 0.05.

*HCC* hepatocellular carcinoma, *APHE* arterial phase hyperenhancement.

### 3.4 ADC value diagnostic performance analysis

The consistency analysis of the ADC values of two radiologists shows that the Kappa value is 0.83, which indicates a high level of agreement between them. For our final analysis, we will use the average of the ADC values measured by the two radiologists as the data.

**Figure 4:**
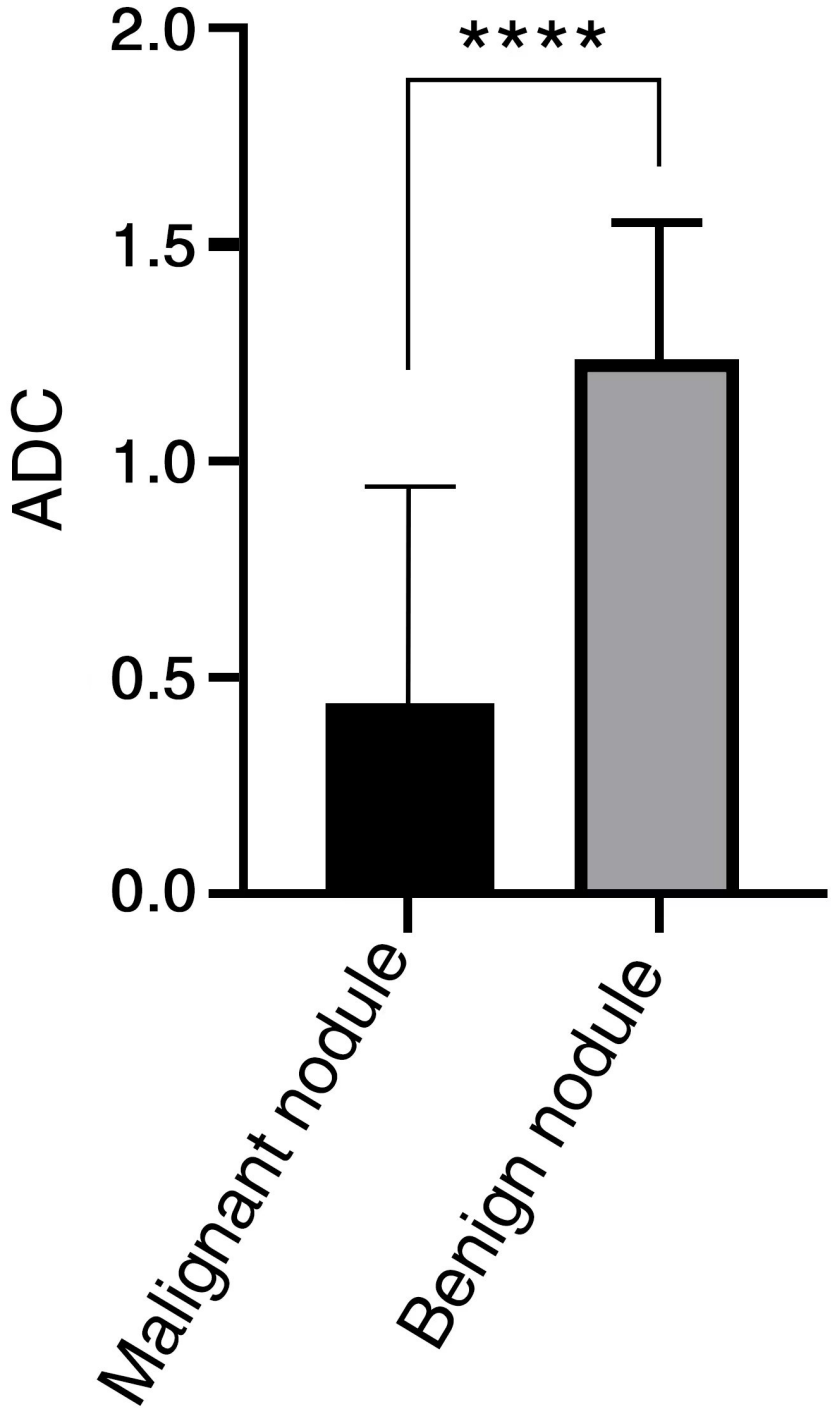
ADC value has significant difference between benign and malignant nodules in LR-3 classification. **** < 0.0001.

The mean ADC values of malignant lesions were 1.01 ± 0.15 × 10^−3^ mm^2^/s and benign lesions were 1.41 ± 0.31 × 10^−3^ mm^2^/s, and the ADC values of malignant tumors were significantly lower than those of benign tumors, and there was a significant difference in ADC values between benign and malignant nodules, p < 0.0001, which was statistically significant (**Figure 4**). ROC analysis was used to analyze the differential diagnostic efficacy of malignant nodules and benign nodules, with an AUC value of 0.909 (95% CI: 0.860 – 0.959), a sensitivity of 92.6%, and a specificity of 74.1%, and ROC analysis is shown in **Figure 3** ②. The optimal threshold for differentiating benign and malignant lesions based on the Youden index was determined to be 1.21×10^−3^ mm^2^/s.

Except for diffusion restriction, which was not significantly different from ADC values, the other ancillary features were significantly different from ADC values. Our statistical results are presented in **Supplementary Table 2**.

## 4 Discussion

In our study, the diagnostic value of ADC values in benign and malignant liver nodules classified by the 2018 version of the LI-RADS criteria as LR-3 classification was investigated. ADC values, nonrim APHE, mild-moderate T2 hyperintensity, and restricted diffusion showed the ability to distinguish malignant from benign nodules in LR-3 classification, with ADC values having the best diagnostic effect (AUC = 0.909), the highest sensitivity, and the high specificity. The results of our study showed that ADC values had a higher sensitivity in discriminating HCC diagnosis in the observation of LR-3 nodules and had a high diagnostic value. Therefore, the application of ADC values in LI-RADS should be emphasized in clinical practice. Among the main features of HCC and other auciliary features, nonrim APHE, mild-moderate T2 hyperintensity, and restricted diffusion were independent factors for predicting HCC, which tended to be consistent with the results of observers of imaging features in other studies’ LI-RADS classification (24–27). Among them, restricted diffusion is close to the AUC value of ADC value, and has a low misdiagnosis rate (specificity 91.2%). Restricted diffusion is judged as the subjective impression of observers, while ADC value is an objective numerical elaboration result of restricted diffusion, and has a high sensitivity. Therefore, ADC value can more objectively reflect the prediction of HCC.

In patients at high risk of HCC, it is difficult to characterize some nodules with unclear HCC image characteristics on contrast-enhanced MRI, especially when the lesions are smaller and classified as LR-3 in the LI-RADS classification, DWI/ADC helps us to observe the lesions (12, 28). In our study, there was a correlation between ADC values and LI-RADS classification, and the mean ADC values of malignant tumors in LR-3 classification were significantly lower than those of benign tumors, and the results tended to be consistent with those reported by Saleh, G.A et al. (29). According to other literature, using DWI/ADC as the main criterion for auxiliary diagnosis improves the sensitivity of diagnosing HCC without significantly reducing the specificity, consistent with our findings (12, 30). Zhong, X. et al. investigated that ADC values improved the ability to distinguish small hepatocellular carcinomas from benign nodules in LR-3 and LR-4 (31), with higher sensitivity and specificity than our findings, possibly due to the exclusion of ICC and cHCC-CC cases from their study samples, which contained cases, and the overlap of ADC values of these lesions with ADC values of HCC may lead to errors in the results. In addition, despite the advantages of ADC values in differentiating benign from malignant tumors, HCC does not differentiate from other malignancies, such as ICC, cHCC-CC, in LI-RADS classification as LR-3.

Limitations remain in this study, first, this is a pilot study performed in a single center and further multicenter studies with larger samples are needed to validate its clinical application. Second, some malignant lesions in this study were obtained by puncture and may have errors due to the heterogeneity of the lesions. Therefore, selectivity deviations cannot be excluded. In addition, the application of hepatobiliary specific contrast agents can make LI-RADS classification more accurate for HCC diagnosis (6), but this examination was not carried out in this study.

## 5 Conclusion

In summary, ADC values showed a high sensitivity for nodules in LR-3 classification while maintaining a high specificity, and ADC values combined with diagnostic criteria from the 2018 version of the Liver Imaging Reporting and Data System (LI-RADS-v2018) could effectively improve the malignant nodule detection rate in LR-3 classification and reduce invasive examinations such as patient follow-up and needle biopsy.

## Data availability statement

The raw data supporting the conclusions of this article will be made available by the authors, without undue reservation.

## Ethics statement

This study was approved by the Ethics Committee of Eastern Hepatobiliary Surgery Hospital, the Third Affiliated Hospital of Shanghai Naval Military Medical University, China, and waived the requirement of obtaining written informed consent.

## Author contributions

Conceptualization, NJ and QC; methodology, XC; software, JX; validation, HH, ZL, and KS; formal analysis, XC; investigation, JX; resources, QC; data curation, XC; writing—original draft preparation, XC; writing—review and editing, WL; All authors have read and agreed to the published version of the manuscript.
