# Supplementary material for: Liver Imaging Reporting and Data System (LI-RADS) v2018: differential diagnostic value of ADC values for benign and malignant nodules with moderate probability (LR-3)
Source: Front Oncol. 2023 Aug 22;13:1186290. doi: 10.3389/fonc.2023.1186290 (PMC10478080; doi:10.3389/fonc.2023.1186290)
Supplement: Supplementary file 1 [file Table_1.docx]

Supplementary Table 1. MRI acquisition parameters

| Sequence | TR/TE  (ms) | Field of view (mm) | Slice thickness (mm) | Slice gap (mm) | Matrix |
| --- | --- | --- | --- | --- | --- |
| T2WI | 2000-8000/80-150 | 380 × 380 | 6 | 1 | 512×512 |
| DWI | 3000/52.4 | 380 × 380 | 5 | 1 | 256×256 |
| T1WI | 3.7/1.85 | 360 × 360 | 6 | 1 | 512×512 |
| LAVA-Flex dynamic | 3.7/1.85 | 360 × 360 | 6 | 1 | 512×512 |

.DWI = diffusion-weighted imaging, LAVA = liver acquisition with volume acceleration. Repetition time = TR, Echo time = TE

Supplementary Table 2. DeLong test

|  | tumor size | nonrim APHE | nonperipheral “washout” | enhancing “capsule” | mild-moderate T2 hyperintensity | restricted diffusion | fat in mass | nodule-in-nodule architecture |
| --- | --- | --- | --- | --- | --- | --- | --- | --- |
| P | <0.0001 | <0.0001 | <0.0001 | <0.0001 | <0.0001 | 0.5625 | <0.0001 | <0.0001 |
| Z | 6.364 | 5.202 | 10.884 | 13.615 | 4.333 | 0.579 | 16.42 | 14.3 |

APHE=nonrim arterial phase enhancement

| Malignant nodule | Number |
| --- | --- |
| **HCC** | 49 |
| macrotrabecular | 30 |
| microtrabecular | 11 |
| sclerosing pattern | 1 |
| pseudoglandular pattern | 1 |
| macrotrabecular-microtrabecular | 4 |
| macrotrabecular massive | 2 |
| **ICC** | 3 |
| **cHCC-CC** | 2 |
| Total | 54 |

Supplementary Table 3.Pathological classification of malignant nodules

HCC=hepatocellular carcinoma , ICC=intrahepatic cholangiocarcinoma , cHCC-CC=combined hepatocellular -cholangiocarcinoma
